# Supplementary material for: Are carbon emissions trading and green financial instruments synergistic? -Comprehensive quantitative research based on content analysis
Source: PLoS One. 2024 Mar 7;19(3):e0298601. doi: 10.1371/journal.pone.0298601 (PMC10919601; doi:10.1371/journal.pone.0298601)
Supplement: S1 Data — (ZIP) [file pone.0298601.s001.zip › Data/地级市Z_F工作报告.zip!/╕╩╦α/╞╜┴╣╩╨/└┤╫╘╣1⁄2╓┌║┼í╛┬φ┐╦╩2╛▌═°í┐_2013.docx]

各位代表：

现在，我代表市人民政府向大会作工作报告，请予审议。并请政协委员和列席人员提出意见。

2012年工作回顾

2012年，在市委的坚强领导下，在市人大、市政协的监督支持下，我们紧紧依靠全市人民，坚持以科学发展观统领全局，围绕落实市三次党代会确立的战略目标，牢牢把握稳中求进、好中求快的总基调，努力克服市场形势复杂多变、国内经济增速放缓带来的严重影响，强力推进重大项目建设，深入开展“双联”行动，集中突破重点工作，较好地完成了市三届人大一次会议确定的目标任务，迈出了转型跨越、科学发展的坚实步伐。预计全市生产总值完成325亿元，比上年增长14%；大口径财政收入完成42.3亿元，地方财政收入完成19.56亿元，分别增长17.1%和24%；城镇居民人均可支配收入达到15425元，农民人均纯收入达到4210元，分别增长15.5%和17.6%。

全力实施“十大工程”，固定资产投资强劲增长。坚持把抓项目、增投资作为促进转型、加快发展的重要支撑，健全完善重大项目推进机制和督查考核办法，全力以赴抓进度、抓质量，全市共实施500万元以上项目893项，其中亿元以上125项，十亿元以上7项，分别比上年增加39项和3项。尤其是精心谋划和组织实施了能源开发、现代农业“五个百万”增收、平凉工业园区基础设施配套、旅游产业提质等“十大工程”，涉及项目109个，年内开工98项，已建成56项，完成投资占年度计划的130.2%。省市列68个重大项目中，中卫至贵阳输气管道（平凉段）等4项建成，泾渗公路等37项顺利推进，平凉机场等预备项目前期工作取得积极进展。“十二五”谋划的14个过百亿元项目开工2项。依靠项目支撑，全市固定资产投资首次突破400亿元大关，达到420亿元，比上年增长44%，投资对经济发展的贡献率达到60%。

突出能源综合开发，工业转型升级步伐加快。以实施能源开发工程为重点，狠抓工业项目建设，全面落实保增长各项措施，支持重点骨干企业加快发展，积极扶持小微企业，工业结构不断优化，全市规模以上工业增加值迈上百亿元台阶。能源化工基地建设进程加快，灵台南部煤田精查结束，灵台独店、泾川高平煤炭资源勘探进展顺利，红河油田百万吨产能会战全面推开，赤城、五举煤矿开工建设，邵寨煤矿建设及百贯沟煤矿改扩建进展良好，20万吨聚丙烯、70万吨烯烃和330千伏灵台送变电工程有序推进，唐家河、南川河煤矿、陇能公司煤转化循环经济等项目前期工作取得积极进展。新兴产业发展势头良好，2.8兆瓦光伏发电示范项目建成，碳纤维产业园一期和光伏产业园一期厂房建设、设备购置抓紧进行。装备制造、农产品加工、新型建材业持续扩容增效，虹光真空接触器技改、静宁纸箱包装产业园和恒达废纸综合利用项目建成，红峰减温减压装置研发生产线投入试生产，亨达公司出城入园和切纸机产业升级改造基本完成，天纤棉业5万锭棉纱生产线、皇甫谧制药公司改扩建完成年度计划。组织实施了一批技术创新项目，开发新产品20项。深入推进清洁生产和节能减排，实施工业节能和循环经济项目21项，完成污染减排项目45个，淘汰落后产能50万吨，单位生产总值能耗降低3.2%，主要污染物排放控制在目标值以内。

大力实施“五个百万”工程，农业农村经济提质增效。围绕促进粮食增产、产业增效、农民增收，集中实施百万吨粮食丰产、百万头肉牛增效、百万亩果园提质、百万吨蔬菜保障、百万亩马铃薯增收工程，推广以全膜覆盖为主的旱作高效农业技术184.6万亩，粮食生产实现“七连增”，总产达到110.35万吨；新建标准化养牛小区56处，冻配改良20.4万头，牛饲养量、出栏量分别增长2.3%和2.2%；新植果树经济林29万亩，创建果园标准化生产基地118万亩，果品总产量95万吨，增长4.5%；新发展设施蔬菜1.16万亩，蔬菜总面积79.8万亩，总产量106万吨，增长5.6%；种植马铃薯100.6万亩，产量达到160万吨，分别增长21.3%和22. 3%。创建省级现代农业示范区2个，新增、规范各类农村专业合作经济组织120个，流转耕地、林地42.8万亩。举办了中国苹果年会暨中国·平凉金果博览会，特色农产品品牌效应日益凸显。完成农民职业技能培训5.26万人、技能鉴定1.8万人，输转农村劳动力47万人，创劳务收入52亿元。认真落实新十年扶贫开发《纲要》，编制了全市六盘山片区区域发展与扶贫攻坚《规划》，实施了39个整村推进、5个整乡推进和华亭黑河流域省级片区扶贫开发项目，易地扶贫搬迁1145户5788人，全市完成减贫10%。兑付各种惠农补贴资金11亿元，人均567元。集体林权制度综合配套改革走在全省前列，市级政策性农业保险补贴试点工作启动。大力改善农村基础条件，完成病险水库除险加固10座，治理河堤90公里，实施农村环境集中连片整治项目54个，土地整理复垦新增耕地1015.2公顷，造林绿化21.1万亩，新修梯田25.2万亩。

激发旅游商贸活力，第三产业发展提速。投资5.8亿元实施重点旅游项目39项，启动了崆峒山生态文化旅游示范区建设。成功举办了第六届世界养生大会、首届皇甫谧故里拜祖大典暨《针灸甲乙经》学术思想国际研讨会、第九届中国民间艺术节暨崆峒文化旅游节等大型节会，在国内外高端平台开展集中宣传推介，崆峒山创建为首批“全国旅游标准化示范单位”，“神奇崆峒·养生平凉”品牌影响力进一步扩大，中远程客源明显增多，全市实现旅游综合收入35.3亿元，增长39.4%。加快商贸物流设施建设，世博伟业家居建材广场、仁河汽配城一期建成运营，风尚吉伍商贸建材市场、平凉工业园区物流中心开工建设，平凉城区建成“放心粮店”20个，万村千乡市场工程覆盖85%的乡镇和70%的行政村。积极实施现代服务业骨干企业培育工程，现代物流、中介服务、住宿餐饮、家政服务等生产生活性服务业较快发展，城乡消费日趋活跃，社会消费品零售总额增长18%。全市第三产业增加值突破百亿元，增速比上年提高2.4个百分点。

深入推进开放创新，招商引资实现新突破。着眼破解资金瓶颈制约，制定落实加快金融业发展的《意见》和金融支持“三农”、支持小微企业发展的措施，切实加强政银企对接，创新拓宽融资渠道，与省农行、省建行、省中行和甘肃银行签订了389亿元的战略合作协议，金融机构贷款余额新增24.5亿元。惠民公司和泓源公司年内融资6.3亿元。新增融资担保机构9户，担保资本金总额达到6.6亿元。改进和加强财税工作，强化税收征管，全市财政收入超额完成了预算任务，争取资金总额增长21.3%，财政支出达到124亿元，增长21.6%，在全省率先完成了规范化乡镇财政所建设。改革理顺平凉工业园区管理体制并启动了新一轮发展规划修编，全市工业园区（集中区）基础设施建设不断加强，年内新入园千万元以上项目31项。全方位推动开放交流与经贸合作，创新招商引资责任落实机制，积极承接东中部产业转移，成功引进陕西星王、甘肃利友、江苏悦达、山东润翔等一批大型企业投资煤电化产业开发和城市基础设施建设，全年实施招商引资项目210项，落实到位资金195亿元，增长83.1%。目前，已有6户世界500强企业、11户中国500强企业和2户民营500强企业在我市投资建设项目69项，招商引资呈现出规模扩张、领域拓展、结构优化的良好态势。着力打造区域性中心城市，城镇化步伐不断加快。理顺平凉城区建设管理体制，拉开“一中心两园区”城市发展框架，编制中心城市相关规划41项，开工建设重点项目8类89个，完成投资25.7亿元。依法实施房屋征收1600多户，征用土地6408亩，有效保障了重点工程建设用地。热电联产集中供热工程完成供热机组热源改造和盘旋路以东主管网敷设，供热面积达到100万平方米。实施了柳湖公园、新区绿地公园一期和一批重点街路绿化及景观提升工程，污水处理厂完成升级改造。312国道城区过境段改线和平定高速西出口连接线工程建成通车，双桥路泾河大桥、东大门道路、平沿路改造等道路畅通工程和“八沟一河”综合治理进展良好，城市服务功能和承载能力有效增强。强化规划、建设执法，集中开展“拆违”和市容环境专项整治，城市管理进一步加强。6县县城和36个重点小城镇实施基础建设项目141项，完成投资12亿元。推进城乡统筹发展，省列泾川新农村试点县、市列3个城乡一体化试点乡镇以及市县列61个新农村试点村、127个“三清五改”示范村建设成效明显。全市城镇化率提高到36.2%。

加快文化改革发展，各项社会事业全面进步。完成了市级文化广电行政责任主体合并及国有文艺院团、电影公司转企改制，广电网络整合运营。市广电新闻中心建成主体，乡镇、社区体育健身中心、城市数字影院和广播电视村村通、村村响工程完成年度任务，“平凉文化年”活动深入开展，《平凉地区志》正式出版。培育文化企业38家，全市文化产业增加值增长28%。教育布局结构调整稳步推进，教育经费保障力度进一步加大，新建、改建中小学校、幼儿园139所，消除D级危房9.5万平方米，18.5万农村中小学生享受到营养餐。市妇幼保健院建成投用，改扩建县级医院5个、乡镇卫生院7个，新建标准化村卫生室31个，建成市、县“120”急救调度指挥系统，为乡镇卫生院配齐了急救车辆，国家基本药物制度实现了基层医疗卫生机构全覆盖，新农合报销总金额、实际住院补偿比分别提高25%和8.5%。人口和计划生育管理服务水平进一步提高，全国利益导向政策体系示范区创建积极推进。信息技术推广应用步伐加快，宽带入户工程进展良好。统计调查、物价、档案、外事、侨务、地震、气象、人防、红十字、残疾人事业等取得新的成效。

加强和创新社会管理，社会秩序保持稳定。强化社区基础设施建设，建成城市标准化示范社区8个。认真落实信访联席会议、领导包案和通报谈话制度，深入排查调处矛盾纠纷，一批重点信访案件得到妥善处理。加强基层综治组织和社会管理基础工程建设，强化流动人口、“两新组织”服务管理和网络信息监管，严厉打击违法犯罪活动，社会治安秩序稳定。加强隐患排查治理和安全生产标准化建设，集中开展“打非治违”专项行动，安全生产形势持续好转。深入推进质量振兴，产品质量、工程质量、服务质量和环境质量不断提升。积极推进药品餐饮食品安全示范县（区）创建，创新完善监管网络和工作机制，强化专项整治，群众饮食用药安全得到有效保障。健全完善应急管理体系，市、县救灾物资储备库全部建成，防灾减灾工作有力有效。双拥共建活动深入开展，崆峒区“两个共同”示范区建设全面推进，全市军政军民团结、民族团结、宗教和顺、社会安定的良好局面进一步巩固。

积极措办惠民实事，民生保障水平不断提升。统筹推进城乡就业，应届高校毕业生就业率达到88%，全市城镇新增就业4.05万人，城镇登记失业率3.6%。城乡居民养老保险制度实现全覆盖，完成了新农合、城镇居民医疗保险补助提标和城乡低保、农村五保供养提标，覆盖城乡的社会保障体系全面建成。市社会福利院建成主体，3个县级社会福利中心投入使用。全市筹资3.1亿元，落实了机关事业单位工作人员、离退休人员津贴补贴和高龄补贴增资政策。省市确定的实事全面完成，开工建设保障性安居工程4246套，主体竣工率达到81.3%；平凉城区9个片区棚户区改造全力推进；完成农村危房改造2.1万户，累计有1.05万户农民搬出窑洞和地坑庄子，基本实现了“告别窑洞”目标。建成农村饮水安全工程19处，解决了15.9万人饮水安全问题，农村自来水通村率、入户率分别达到83.2%和82.5%。实施县乡道路改造5条134公里，新建通村硬化路474公里，开通城乡公交班线12条。投资1100万元更新平凉城区公交车28辆，新建公厕10座，中心城市智能交通信号灯及指挥系统建成投用，乡镇视频监控系统建设进展顺利。建成户用沼气池1万口、村级服务网点50处，为5450户农户配发了太阳灶。全市财政用于民生的支出占到总支出的73.3%。

一年来，我们适应加快转型跨越、促进科学发展的任务要求，积极推进政府职能转变和管理创新，建立健全了市级财政投资项目管理、重大项目安排和大额度资金使用等制度，修订完善了县（区）、部门责任目标考核办法，制定规范性文件14件，政府规范化、科学化决策管理水平不断提升。自觉接受人大法律监督、工作监督、政协民主监督和社会舆论监督，积极配合人大、政协开展视察、调研，高度重视人大代表建议和政协提案办理，183件建议提案均按期办结。按照省、市委开展“双联”行动的统一部署，各级领导带头，广大干部积极参与，深入开展进村入户走访、政策技术宣讲、矛盾纠纷排查、示范典型培育和助农增收帮扶工作，规划实施产业帮扶项目482个，完成投资2.5亿元，联系乡村的生产生活条件明显改善，群众收入有较大提高，机关干部的群众观念进一步增强，工作作风有效转变。深入开展效能风暴行动，取消和调整行政审批事项64项，取消行政事业性收费26项，降低收费标准70项。全面推进依法行政和政务公开，加快电子政务建设和办公无纸化，基本实现了部门间公文电子化传输。加大纠风治乱和民主评议行风力度，加强政府投资项目、社保资金和经济责任审计，强化行政效能监察和廉政风险防控，政府系统反腐倡廉建设深入推进。

各位代表，一年来，全市经济社会发展取得的成绩来之不易，这是省委、省政府和市委坚强领导的结果，是市人大、市政协监督支持的结果，更是全市干部群众共同努力的结果。在此，我代表市人民政府，向辛勤工作在各条战线的广大干部群众，向中央、省驻平单位和驻平部队，向各民主党派、工商联、无党派人士，向离退休老同志，向关心、支持平凉建设和发展的各界人士，致以崇高的敬意和衷心的感谢!同时，我们也清醒地看到，全市经济社会发展和政府工作中还存在不少困难和问题，主要是：经济总量小，项目建设总量不足、规模偏小，尤其是支撑和带动跨越发展的大项目、产业项目不多；经济结构不够合理，工业结构比较单一、产业链条短，非公有制经济发展缓慢、比重小，现代服务业规模、层次较低；农业基础脆弱，产业化程度不高，蔬菜等农产品供给保障不足，农民持续增收的难度大；资金、人才和基础设施瓶颈制约仍然突出；就业形势依然严峻，部分低收入群众生活比较困难，公共服务水平与人民群众的愿望仍有较大差距；一些部门、单位和干部创新意识、服务意识不强，工作起点不高，推动落实不力，庸懒散慢浮的问题还比较突出。对此，我们将认真分析研究，采取切实可行的措施加以解决。

2013年工作要求和主要任务

2013年是全面贯彻落实党的十八大精神的开局之年，是实施“十二五”规划承前启后的关键一年。十八大描绘了全面建成小康社会的宏伟蓝图，开启了改革开放和现代化建设的新征程，提出优先推进西部大开发、促进区域均衡发展等重大战略举措，为我们推动科学发展、加快小康进程提供了难得机遇；关天经济区、六盘山片区扶贫攻坚、陕甘宁革命老区振兴等规划的深入实施，为我们夯实发展基础、实现后发赶超提供了政策支持；东中部地区资源型、高载能产业向西部地区加速转移，为我们集聚域外资源、扩大开放开发提供了有利条件；全省区域发展战略的深入推进和“3341”项目工程的全面实施，为我们扩大项目总量、加快产业升级提供了强大动力。同时，我们面临的宏观经济环境更加复杂，要素制约更加突出，市场和区域间竞争愈加激烈。新的形势、新的机遇、新的挑战，需要我们千方百计加快发展步伐，持续不断提升发展质量，坚定不移推进经济社会转型跨越、科学发展。

政府工作的总体要求是：深入学习贯彻党的十八大精神，坚持以邓小平理论、“三个代表”重要思想、科学发展观为指导，全面落实中央经济工作会和省委全委扩大会、省十二次党代会、市三次党代会的决策部署，紧盯全面建成小康社会目标，围绕主题主线，坚持工业化、信息化、城镇化、农业现代化同步发展，深入实施全省“十大行动”和“3341”项目工程，全力保增长、转方式、调结构、强产业、夯基础、促和谐，推动项目建设跃上新台阶，产业升级迈出新步伐，城乡面貌发生新变化，改善民生取得新成效，切实加快小康和谐文明生态平凉建设进程。

经济社会发展的主要预期目标是：生产总值增长13.5%，规模以上工业增加值增长16%，固定资产投资增长35%，地方财政收入增长16%，社会消费品零售总额增长18%，城镇居民人均可支配收入增长16%，农民人均纯收入增长16%，城镇登记失业率控制在4%以内，人口自增率控制在7.1‰以内，单位生产总值能耗和主要污染物排放控制在省上下达目标之内。

围绕上述目标，按照“提速进位，重点突破，改革创新，抓实见效”的要求，重点抓好以下六个方面工作：

（一）强力推进项目建设，持续做大经济总量。大力实施项目带动行动，继续抓好“十大工程”，精心谋划和实施一批能够扩大投资、加快转型、优化结构、改善民生的重大项目，以大项目拉动大投资、促进大发展。

加快重大基础设施项目建设。着眼破解瓶颈制约，完善交通路网框架，完成泾渗二级公路、崇白路改造，力争西平铁路建成通车，加快天平铁路建设，完成平天高速、静庄高速、彭阳至大桥村高速和312国道平凉段改造前期工作并争取尽早开工，促进平凉机场早日立项。强化水利保障，编制全市水资源可持续利用规划，积极配合做好引洮二期静宁受水区工程、白龙江引水平凉支线工程前期工作，促进崆峒水库改扩建等骨干水源工程前期工作取得突破性进展；完成泾川泾庆灌区和静宁西干渠续建配套与节水改造，建成一批重点河流防洪工程。加大信息化基础设施建设投入，加快3G移动互联网、数字城市、数字企业、数字社区建设，扩大信息技术在新农村建设、城市管理、社会管理及生产服务等领域的应用，推动信息化和工业化深度融合。

加强能源化工项目建设。围绕建设国家级能源化工基地，快速推进邵寨、赤城、五举煤矿和红河油田百万吨产能建设，加快中煦20万吨聚丙烯、华泓汇金180万吨甲醇及70万吨烯烃建设进度，开工建设陇能公司360万吨兰炭、42万吨焦油加氢项目及唐家河煤矿，南川河、安家庄煤矿完成前期工作并力争年内开工，天元公司煤电化循环经济项目取得实质性进展。加快输电通道和联网工程建设，建成330千伏灵台送变电工程，开工建设110千伏米家湾、崇信送变电工程，加快330千伏尚家塬二期前期工作，提升供电保障和电网资源配置能力。

提高项目谋划争取和保障服务水平。深入论证对接，加大项目储备，优化项目结构，提高非资源类、高新技术类、民生类项目比重，全市储备项目投资额动态保持在2000亿元以上，其中工业项目投资额占到一半以上。加大经费投入，做深做细前期工作，强化衔接争取，力争有更多项目挤进国家和省上先期实施计划，扩大投资增量。优化政府服务，加强协作配合，提高申报、审批效率，做好土地供应、设施配套、资金保障等协调服务，严格施工管理和质量监管，优质高效推进各类项目建设。

（二）加快转变发展方式，促进产业优化升级。以构建现代产业体系为方向，大力调整产业结构，做大做优特色优势产业，改造提升传统产业，加快培育战略新兴产业，支持发展现代服务业，着力提高经济发展质量和效益。

持续推进工业转型升级。以打造千亿元煤电化冶产业集群为目标，加大资源勘探开发力度，完成灵台独店、泾川高平煤炭资源精查，实施泾川东部、崆峒北部、华崇交界三大区块煤炭资源普查详查，加快矿井、油田、精细煤化工项目建设，延伸产业链条，协调支持煤炭、电力等骨干企业提高产量，做大做强区域首位产业，全市生产原煤2600万吨，发电量达到200亿千瓦时。建成光伏产业园、碳纤维复合材料园一期工程并启动二期建设，开建生物胶原蛋白肽生产线，推动新兴产业发展取得新突破。改造提升装备制造和建材业，做精做优“阀、管、刀”等主导产品，积极推进临工工程机械、华星公司专用车改装、华伟公司摩托车总装生产线等项目，建成庆华公司1600万平方米建筑陶瓷、永兴公司50万立方米轻质墙体材料、天福公司高档门窗生产线。实施一批技术创新项目，完成新产品研发20项。

加快发展现代农业。继续实施现代农业“五个百万”增收工程，强化科技带动，突出规模化养殖、品种改良和饲养方式转变，牛饲养量、出栏量分别达到110万头和41万头；加快果产业扩量提质增效，新植果园28万亩，巩固提升标准化基地100万亩，果品总产量达到100万吨以上，新增果品贮藏能力5万吨；发展设施蔬菜1.1万亩，蔬菜总产量达到100万吨以上，马铃薯脱毒种薯应用面积达到100万亩，推广旱作高效农业技术240万亩，粮食总产稳定在100万吨以上，确保粮食安全。建成正大30万吨饲料一期生产线、静宁金果国际博览城等农业产业化项目，新建、改扩建龙头企业20户，创建省级现代农业示范区2个，新增、规范农民专业合作社50个。持续推进新农村建设，积极争取实施省列新农村试点县项目，抓好市列3个城乡一体化乡镇、20个新农村试点村和100个“三清五改”示范村建设。

壮大提升第三产业。强化旅游商贸带动，集中实施崆峒山景区旅游服务扩容、南石窟寺景观大道等重点旅游项目，加快景区、饭店晋等升级，支持开发养生产品，延伸养生产业链条，完善提升旅游要素，持续扩大宣传推介，全年游客接待量和旅游综合收入增长20%以上。开工建设平凉客运枢纽站，加快平凉工业园区物流中心、陇东农产品批发交易中心、风尚吉伍商贸建材市场及仁河汽配城二期等大型商贸设施和物流配送中心建设，实施万村千乡市场工程，支持批发、零售骨干企业发展电子商务，活跃城乡消费。积极拓展领域，促进现代服务业与特色优势产业有机融合，加快服务产品、服务模式创新，推进现代物流、金融、科技、信息等生产性服务业和商贸、文化、保健、养老、家政服务等生活性服务业快速发展。全市第三产业增加值增长13.5%。加强农产品出口基地和重点出口企业规模化建设，扩大对外贸易，全市进出口总额增长10%。

积极发展循环经济。认真实施循环经济总体规划，着力推进绿色发展、循环发展、低碳发展，抓好华亭工业园区省级循环化改造，搞好省级循环经济示范园区、示范企业创建。深入推进节能减排，实施平凉电厂2台机组脱硝改造等一批节能技改和污染减排项目，严格高耗能、高污染项目审批管理，促进节约农业、循环农业发展，强化水、大气、土壤等污染防治和农村环境集中整治，切实解决影响群众健康的突出环境问题。积极推进国家生态市创建，实施好泾渭河流域百万亩生态防护林基地等重点林业生态工程和水土流失治理工程，抓好城镇面山、道路沿线绿化和生态修复，加快生态文明建设。

（三）实施中心城市带动战略，增强聚集发展能力。坚持工业化和城镇化良性互动、城镇化和农业现代化相互协调，大力推进“一中心两园区”建设，扩大中心城市规模，提升公共设施服务能力，改善人居环境，促进人口转移、产业集聚，加快建设甘肃东部门户城市。

打好中心城市建设攻坚战。按照轴线式推进、组团化开发的思路，着力完善城市功能，提升城市形象，带动全市城镇化水平提高2.5个百分点。突出规划引领，完成第四轮《平凉市城市总体规划》修编，修订、完成相关街景规划和修建性详规，提升规划层次，强化规划执法。着力打造以行政中心为主要标志的城市中轴和崆峒大道、泾河两岸三条景观带，集中实施“四大组团”，建设以平沿路、泾河水景工程、休闲度假区为重点的生态文化旅游组团，以绿地公园、党政群机关统办楼、商业综合体为重点的行政商务组团，以双桥路北拓、柳湖路东拓、东大门道路改造等为重点的城东枢纽组团，以平镇路改造、南北路泾河大桥为重点的工业园区基础设施配套组团。全力抓好“六大工程”建设，加快推进热电联产工程，集中供热面积达到400万平方米以上，覆盖面达到70%以上；实施保障性住房、6个片区棚户区改造和6处村民安置楼建设工程；推进城区污水收集、道路维护、“八沟一河”综合治理等9项城市基础配套工程；抓好城区学校、环卫设施、停车场、殡仪服务中心等13项城市功能完善工程；实施城区重点路段亮化、高速公路两侧绿色通道等3项城市品质提升工程；实施15项房地产综合开发工程。强化城市管理，健全长效机制，加大综合执法力度，提高城市规范化管理水平。积极开展园林县城、生态宜居小城镇创建，配套完善道路、排污、供热等基础设施，提升县城、小城镇容纳人口、吸纳就业能力和产业发展水平。

大力推进工业园区建设。围绕打造国家级经济技术开发区，以在全市率先建成小康社会、率先实现城乡一体化为目标，加快平凉工业园区基础设施建设，完成铁路运输专线、供水管线、物流站场三大配套工程前期工作，健全园区服务功能，完善管理运行机制，扩大投资强度，年内新入园千万元以上项目20个，其中亿元以上项目力争达到10个，固定资产投资达到105亿元。启动实施华亭、静宁工业园区体制改革，抓好各类工业园区（集中区）规划、建设和项目入园，强力拓展园区承载能力和发展外向度。

加快崆峒山生态文化旅游示范区建设。围绕打造“神奇秀美崆峒山，天下养生第一地”，深入挖掘崆峒山文化内涵，高标准完成崆峒山生态文化旅游示范区规划编制，开工建设崆峒山生态旅游度假山庄，完成崆峒古镇二期、崆峒山问道宫景观园前期工作，加快建设人文生态、综合服务、产品研发、休闲度假、民俗风情、文化养生六大示范基地，推动文化、生态与旅游深度融合。积极创造条件，主动融入全省华夏文明保护传承和创新发展示范区，争取国、省更多项目资金和政策支持。

（四）不断深化改革开放，切实增强发展活力。坚持激活内力与借助外力并重，在深化改革中破解发展难题，在扩大开放中拓展发展空间。

全方位扩大招商引资。完善招商引资工作推进机制和考核办法，调动各级各部门和广大干部的积极性、创造性，营造重招商、招大商、服务招商的浓厚氛围。创新招商方式，紧盯重点产业、重点企业和重点区域，多渠道、多层次引进一批财源型、生产型、高新技术型和就业容量大的项目。加强社会信用体系建设，落实招商引资优惠政策，强化政府服务，畅通招商项目建设绿色通道，吸引更多企业和项目落户。加强招商引资项目调度管理，抓好签约项目的对接落实和跟踪服务，提高项目履约率、资金到位率和开工投产率，招商引资到位资金增长35%以上。

推动非公有制经济跨越发展。认真落实各项扶持政策，强化指导服务，鼓励支持全民创业，大力提升非公有制经济发展的层次和水平。放宽市场准入，激活民间资本，鼓励支持非公经济主体投资发展牛果菜特色优势产业，平等参与矿产资源勘探开发及市政设施、公共交通、保障性住房建设，支持民间资本投资兴办医疗卫生、养老托幼服务等社会公益事业。大力营造亲商、富商、安商的良好环境，搞好各项服务，着力协调解决企业资源配置、投资融资、建设用地等方面困难和问题，建立财政扶持非公经济发展专项资金，支持非公企业加快技术创新和结构调整，推动企业转型升级。大力实施百户小微企业创业帮扶工程，通过重点扶持、招商引资、兼并重组等途径，培育形成销售收入过5000万元非公企业20户，过亿元企业10户，非公有制经济增加值占生产总值的比重提高2个百分点以上。

着力破解资金瓶颈制约。创新加强金融工作，完善政银企联席会议制度，促进项目和金融产品有效对接，争取金融机构贷款余额增长20%以上。积极开发资本市场，培育拟上市资源企业。创设良好环境，促进引行入平，争取兰州银行、省开发银行、交通银行在平设立分支机构。创新融资手段，力争发行企业债券10-15亿元。进一步做大惠民公司和泓源公司，加快组建交通融资实体、水务集团和旅游投融资公司，融资总额力争达到30亿元以上。积极发展小额贷款公司、融资担保公司，探索发展农村资金互助组织。扩大农业政策性保险试点范围，拓宽保险覆盖面。发挥财政资金导向作用，采取贴息、奖励等措施，吸引社会资本向重点产业、重点项目投入。加强财税收入征管，整合项目资金，集中财力办大事。加大资金争取力度，确保转移支付和各类补助资金增长20%以上。

推进重点领域改革。创新政府管理方式，推进招标投标管理和公共资源市场化改革，加快建立统一规范的招投标和公共资源交易市场。全面推行国库集中支付改革和公务卡应用。完成商贸企业改革，妥善解决其他改制企业遗留问题。巩固和扩大基本药物制度改革成果，稳步推进公立医院改革，抓好科技、教育、文化体制和事业单位改革，加快形成政府主导、覆盖城乡、可持续的基本公共服务体系。完成覆盖农村集体各类土地的所有权确权登记颁证工作，深化集体林权制度综合配套改革，加快水利改革试点工作。

（五）着力改善民计民生，提高人民生活质量。坚持民生优先、富民惠民的发展取向，下工夫解决群众最关心最直接最现实的利益问题，努力让人民群众过上更好生活。

积极扩大就业。实施就业优先战略，认真落实促进就业各项政策，加强就业培训和指导，促进高校毕业生、城镇失业人员、特殊困难人员充分就业。加快创建省级创业型城市，推动创业促进就业，帮助3000名自主创业人员实现成功创业。鼓励发展劳动密集型产业、服务业和公益性就业企业，增加就业岗位。加大农民职业技能培训，扩大有组织、技能型劳务输转，增加农民工资性收入。

不断完善社会保障体系。巩固城乡居民社会养老保险参保成果，参保率达到95%以上。全面推进非公有制经济组织和灵活就业人员参加社会保险，稳步提高城镇五项社会保险覆盖率。推进被征地农民、村干部养老保险与城乡居民养老保险并轨，建立失业保险促进就业联动机制。提高城镇低保标准，规划建设一批城乡社会福利设施，完善社会救助体系和优抚安置保障制度。

深入推进扶贫攻坚。加快实施六盘山片区区域发展与扶贫攻坚规划，抓好35个整村推进、6个整乡推进及崆峒区西北部干旱塬区省列片区开发项目。继续抓好崆峒区“两个共同”示范区建设，支持民族乡村加快发展。深入推进“双联”行动，落实帮扶计划，力争贫困地区农民人均收入增长17. 5%，实现减贫20%。

积极措办惠民实事。1．新增城镇就业2.7万人；应届高校毕业生就业率达到80%以上。2．新建保障性住房9949套；实施农村危房改造2万户。3．改造县乡道路100公里；新建通村硬化路450公里；建成行政村停靠站50个，开通城乡公交班线12条。4．新建 28处农村饮水安全工程，解决农村15万人饮水安全问题。5．实施以工代赈易地扶贫搬迁工程，搬迁农村贫困群众1623户8117人。6．新建校舍15万平方米；新建、改建幼儿园44所；实施农村义务教育薄弱学校食堂改造20个、2万平方米。7．在重点小城镇和建制镇建设公租房1130套，改善乡镇基层住房困难职工的住房条件。8．新建标准化村卫生室20个；新建乡镇、社区体育健身中心７个。9．建成农村户用沼气3000户；发放太阳灶5000台。10．建成城市数字影院3座、放映厅9个；实施宝塔梁维修加固和综合开发工程，建设城东文化活动中心。

（六）协调发展社会事业，推进和谐社会建设。以满足群众文化教育、医疗卫生等基本需求为目标，统筹推进各项社会事业快速发展，创新和加强社会管理，切实增强人民群众的安全感、幸福感。

优先发展教育科技事业。启动实施义务教育均衡发展规划，调整优化教育布局，实施平凉一中扩建二期，开工建设平凉第十中学和广成小学。加快静宁教育苑建设。继续实施好校舍安全工程，大力发展学前教育、职业教育，加快平凉医专升本和平凉高等职业技术学院建设步伐。加强校园安全和校车安全管理。开展教育质量管理年活动，提高教育教学质量。实施创新驱动发展战略，着力构建以企业为主体、市场为导向、产学研相结合的技术创新体系，促进科技成果与产业化结合，组建2个市级工程技术研究中心，加强科技企业孵化器建设，确保在孵企业达到15户以上。

加快文化名市建设。加强社会主义核心价值观宣传教育，大力弘扬平凉精神，抓好全国文明城市创建，持续开展多种形式的精神文明创建活动。开工建设市体育中心，建成市广电新闻中心。全面提升县级“三馆”、乡镇文化站、农家书屋服务水平，深入实施文化惠民工程，加强文物和文化遗产保护。抓好生态民俗文化体验区建设，规划建设7个特色文化产业园和一批特色文化街区，发展壮大文化产业，文化产业完成投资6亿元，增加值增长28%以上。

提高医疗卫生服务水平。继续加强县、乡、村和社区医疗卫生机构建设，加大乡村医疗卫生人员培训力度，完善城乡公共卫生服务体系，大力发展中医药事业，健全全民医保体系，完善突发公共卫生事件应急和疾病预防控制机制，提升基本医疗保障水平和公共卫生服务能力。扎实做好人口和计划生育工作，深入推进全国利益导向政策体系示范区创建，统筹解决人口问题，稳定低生育水平。

进一步创新和加强社会管理。重视加强社区建设，合理调整社区规模，充实工作力量，提升服务功能，新建城市标准化社区11个。扩大农村社区建设试点，充分发挥乡村综合治理、人民调解等组织的作用，广泛开展农村“和谐五星”创评活动。坚持用群众工作统揽信访工作，落实信访工作责任制和重点信访案件领导包案、督查督办制度，畅通和规范群众诉求表达渠道，及时有效化解各类社会矛盾。深入开展民族团结进步创建活动，促进宗教事务管理规范化法制化。持续推进平安建设，完善打防管控一体化社会治安防控体系，严密防范和打击各类违法犯罪活动，促进社会和谐稳定。加强市场管理，加大食品、药品、农资等专项整治力度，全面推行食品药品安全城乡一体化监管模式，保障群众消费安全。严格落实安全生产责任，切实强化基层基础工作，有效防范和遏制重特大安全事故，确保安全生产形势稳定。健全完善应急体系，提升突发事件应急、处置能力。

加强政府自身建设

做好今年工作，困难不少，责任重大。我们将把加快发展作为政府工作的根本职责，把群众满意作为政府工作的第一追求，不断加强自身建设，着力提高政府创造力、执行力和公信力，更好地肩负起加快转型跨越、促进科学发展的重任。

解放思想推动发展。把加强学习作为解放思想的首要任务，深入学习贯彻党的十八大精神，强化公务员教育培训，加强政策学习研究，注重从政策条文中捕捉信息、对接项目、寻找资金，真正把政策机遇更多地转化为促进发展的实际举措。把正视差距作为解放思想的内在动力，站在全省全国发展大局中审视自己，自我加压，拼抢提速，争先进位，努力推动更好更快发展。把发展成效作为检验解放思想的标准，坚决摒弃不敢想、不敢干的畏难情绪，按照建成全面小康社会的目标，倒查倒逼，攻坚克难，主动作为，激情干事，形成凝心聚力抓落实，只争朝夕促发展的浓厚氛围。

创新机制促进落实。创新重大项目、重点工作推行机制，建立完善“一名主抓领导，一个责任单位，一套工作班子”的责任体系，明确职责任务，全面提高推动落实的质量和效率。创新绩效考评机制，健全完善经济社会发展目标、项目建设、招商引资、争取资金等考核办法，强化目标责任管理。开展“十二五”规划实施情况中期评估。加强经济运行分析调度，逐月分析、按季调度、及时通报，确保各项工作任务按时限、按节点有序推进。健全重大决策执行情况督查反馈机制，坚决做到令必行、行必果，切实提高政府的执行力。

转变作风提升效能。严格落实中央关于改进工作作风、密切联系群众的八项规定和省、市委各项具体规定，大力弘扬求真务实、真抓实干作风，深入调查研究，解决实际问题，力戒形式主义、官僚主义。切实改进会风文风，少开会、开短会、讲短话，规范减少文件简报，严格控制各类剪彩、奠基活动及纪念会、表彰会、研讨会。深入推进效能风暴行动，深化行政审批制度改革，加强各级政务网站和电子政务建设，推进无纸化办公，尽快建成网上审批和电子监察系统，抓好市政府政务大厅规范运行，推行“一厅式”、“一站式”服务。加强效能监察，严格行政问责，治庸治懒治散，下决心解决办事拖拉、效率低下等突出问题，努力建设高效务实政府。

依法行政规范行为。进一步完善政府议事规则及重大决策咨询、听证、公示、风险评估等制度，提高政府决策的科学化民主化规范化水平。深入推进“六五”普法，提高全民法制观念，增强各级干部特别是领导干部运用法治思维和法治方式深化改革、推动发展、化解矛盾、维护稳定能力。规范政府行为，完善行政执法责任制和过错追究制，解决有法不依、执法不公、多头执法等问题，切实做到严格规范公正文明执法。自觉接受人大法律监督、政协民主监督，及时办理人大代表建议和政协提案，认真做好人大视察、政协调研意见建议的落实和反馈，高度重视社会舆论监督，全面推进政务公开，保障人民群众知情权、参与权、表达权、监督权。廉洁从政树立形象。按照建设廉洁政府的要求，严格落实党风廉政建设责任制，加强廉政风险防控，强化审计监督，健全建筑工程、土地交易、产权交易、政府采购、医药购销、资金监管、国有资产管理等重点领域的反腐倡廉长效机制，严格按程序审批出国（境）考察，加大纠风治乱力度，严肃查处违法违纪案件。倡导艰苦奋斗，厉行节约，压缩非生产性开支，规范公务接待，制止铺张浪费，把有限的资金用在促进发展、改善民生上。

各位代表！新的目标催人奋进，新的征程任重道远。让我们在党的十八大精神指引下，在市委的正确领导下，紧紧团结和依靠全市人民，深入贯彻落实科学发展观，凝心聚力，攻坚克难，真抓实干，锐意进取，为加快转型跨越、促进科学发展，建设小康和谐文明生态平凉而努力奋斗！
